# Increasing Our Understanding of How Dietary Components Can Affect Cellular Mechanisms That Regulate Aging and Slow the Onset of Frailty and Chronic Diseases

**DOI:** 10.3390/nu15122687

**Published:** 2023-06-09

**Authors:** Cristiano Capurso

**Affiliations:** Department of Medical and Surgical Sciences, University of Foggia, Viale Pinto 1, 71122 Foggia, Italy; cristiano.capurso@unifg.it

The current average life expectancy at birth is well over 80 years. Such high longevity in many cases is associated with the onset of chronic diseases and frailty. Cardiovascular disease still represents the first cause of mortality in the population; among the elderly, the most frequent pathological conditions are due to cardiovascular pathologies: for example, hypertension and heart failure.

Maintaining an adequate nutrition status and lifestyle is important, both to reduce the risk of chronic diseases, and to slow or delay the onset of frailty.

Dietary patterns, often considered a lifestyle factor, affect the development of many chronic conditions including obesity, cardiovascular disease, hypertension, stroke, type 2 diabetes, metabolic syndrome, some cancers, and some neurological disorders.

Dietary patterns such as the Mediterranean diet have been shown to reduce cardiovascular risk, to slow the aging process and to delay the onset of frailty, all characterized by chronic low-grade inflammation, by influencing chronic inflammatory processes and responses. To date, the scientific literature has identified robust evidence relating to the relationship between greater adherence to the Mediterranean diet and a reduction in overall mortality and the incidence of cardiovascular disease, as well as the overall incidence of cancer, neurodegenerative diseases, and diabetes [1].

Previous evidence has focused primarily on adequate protein intake being advocated as a possible intervention for the management of frailty in the elderly, by virtue of the protective effects on muscle mass and physical function [2]. Subsequently, other nutrients, in particular carbohydrates and added sugars, were also studied as being responsible for negative effects on the metabolism during aging and as having a decisive role in frailty. Further evidence has shown that an increase in the intake of simple carbohydrates, to the detriment of complex ones in the diet, induces negative effects on aging, as it is associated with a greater risk of frailty [3,4].

The main objective of this Special Issue was to address the existing knowledge on carbohydrate intake and glucose metabolism as causal factors of frailty and aging-related diseases, i.e., to identify strategies to delay the pathological effects of aging.

In their cross-sectional and longitudinal analysis up to 13 years of follow-up, Tanaka et al. showed that higher tertiles of carbohydrate consumption were associated with worse frailty, as measured by the 43-item frailty index. On the contrary, higher consumption of fibers, evaluated as fiber-to-carbohydrate ratio, was associated with a lower frailty index. Particularly in women, a higher ratio of fiber to carbohydrate was associated with slower progression of the frailty index over time, suggesting that a diet rich in fiber can slow the accumulation of deficits related to aging frailty over time [5].

Dong et al., in their cross-sectional study, further reaffirmed the protective role of a higher intake of dietary fiber versus carbohydrate, expressed as carbohydrate-to-fiber ratio (CFR). They showed that higher CFR quartiles, indicative of low dietary fiber versus carbohydrate intake, were associated with poor blood pressure control, compared with lower quartiles, conversely indicative of higher dietary intake of fiber over carbohydrates [6].

Several pieces of evidence have demonstrated the efficacy of dietary fiber intake, both as soluble fibers and as fibers from natural foods, in short- and long-term glycemic control and on insulin response in patients with type 2 diabetes, where the former would produce better effects. That is, the extent of the reduction in blood glucose and insulin response depends on the type of fiber, the dose, and the duration over time of dietary consumption [7]. Crummet and Grosso aimed to further confirm the protective role of fibers, especially soluble fibers, on postprandial glycemic control in a study conducted on a small sample of young, healthy college students, after consuming whole apple and blackberries versus blended apple and blackberry. The postprandial glycemic response, measured as the 30-minute incremental area under the glucose curve, was significantly lower in blended fruit than in whole fruit, while the 60-minute glycemic response was only slightly lower in blended fruit than in whole fruit. The authors hypothesized that this lower glycemic response from the consumption of pureed fruit compared to whole fruit, naturally without added sugars, could be attributed to the higher proportion of soluble dietary fibers absorbable after blending, thus confirming the healthful effects of a high fiber intake [8].

On the other hand, the detrimental effect of a high intake of simple sugars on the aging of organs and systems is well known. It is known that cardiovascular disease mortality increases exponentially with increasing levels of consumption of simple sugars in the diet [9]. An interesting study conducted on animal models (young mice vs. old mice) confirmed that a high intake of simple sugars in the diet is associated with cardiac hypertrophy, especially in old mice. In elderly mice, a greater intake of simple sugars was related to a greater cardiac mass and a greater wall thickness of the left ventricle. Furthermore, a higher intake of simple sugars was associated with an increase, although not significant, of the mitochondrial production of ROS [10].

Numerous pieces of evidence have shown that an adequate regimen of caloric restriction, where it does not lead to a state of malnutrition, produces beneficial effects in terms of slowing down aging and increasing lifespan. Intermittent fasting interventions, by restricting all calorie intake without altering diet quantity and quality, have been found to provide multiple health benefits, including reduction of body weight, fat mass, systolic and diastolic blood pressure values, improvement of insulin sensitivity, as well as extending healthy lifespan, mostly in adults with overweight or obesity [11].

Further, Saini et al. showed that time-restricted eating, a popular form of intermittent fasting, provides multiple health benefits, including an extension of a healthy lifespan, by downregulating the expression of microRNA that mediate the expression of genes associated with the regulation of cell growth and proliferation, protein synthesis, insulin signaling, and autophagy [12].

In conclusion, the studies presented in this Special Issue, as well as in the wider scientific literature in general, contribute to our constantly evolving understanding of how dietary components, and in general, a healthy lifestyle can affect cellular mechanisms that regulate aging and slow the onset of frailty and chronic diseases, especially cardiovascular disease. The evidence presented in this Special Issue may also contribute to helping healthcare professionals educate their patients and facilitate their adoption of healthy eating behaviors.
